# Media portrayal of older people as illustrated in Finnish newspapers

**DOI:** 10.3402/qhw.v9.25304

**Published:** 2014-09-24

**Authors:** Sanna Koskinen, Leena Salminen, Helena Leino-Kilpi

**Affiliations:** 1 Department of Nursing Science, University of Turku, Turku, Finland; 2 Turku University Hospital, Turku, Finland

**Keywords:** Older people, society, attitudes, newspapers, thematic analysis

## Abstract

Media portrayals of older people, such as those in newspapers, both inform and reflect public attitudes. By becoming aware of culturally influenced attitudes about older people, and how these attitudes are reflected in the ways older people are viewed, treated, and cared for in society, the healthcare profession can better understand how to provide high-quality care. By applying an ethnographic approach in textual reality, this paper explores how newspaper articles focusing on health portray older people in society, using Finland as an example. The data consist of articles selected from three of the main Finnish daily newspapers during a 3-month period in the spring of 2012. The findings show that, overall, the society regards older people and their care as important. However, there were suggestions of paternalistic attitudes towards older people. Furthermore, the perceptions regarding different groups of older people could lead to the possibility of inequality. The media portrayals of older people worldwide seem to share similarities, although the findings of this study are particularly in accordance with the cultural attributes of the Nordic countries and societies.

The growing number of older people in many nations has stimulated researchers to study media portrayals of older people (Zhang et al., 2006). These media portrayals have the dual function of both informing and reflecting people’s explicit and implicit attitudes and beliefs about, and the actions they take towards, older people (Fealy, McNamara, Treacy, & Lyons, 2012). First, societies adopt their attitudes by observing and modeling the media portrayals of older people (Bandura, 2002). Second, continued exposure to media portrayals of older people will most likely reinforce, confirm, and nourish people’s values and perspectives so that they match those delivered by the media (Gerbner, Gross, Morgan, Signorielli, & Shanahan, 2002).

Newspapers are an influential part of the media. As an important means of communication they shape our perceptions about the world (Fealy et al., 2012), thus also reflecting the ways older people are treated and cared for in society (Phelan, 2011). Public portrayals are important because they include cultural values, social norms, and role expectations. Knowledge about these portrayals is important because they affect the ways in which health and healthcare practice are perceived in different societies (Kim, 2010; Phelan, 2011). This study addresses the issue of societal attitudes towards older people by exploring how older people are portrayed by newspapers at a time when greater numbers of older people are requiring a wide range of health and social services, because attitudes eventually influence the quality of care provided.

Research into how older people are portrayed in the media has been fairly extensive. These studies have mostly been carried out in the fields of social and communication sciences and gerontology, but seldom in the nursing field. Various forms of media have been of interest: drama television series (Lien, Zhang, & Hummert, 2009; Signorielli, 2004), advertisements on television (Kessler, Schwender, & Bowen, 2010) and in magazines (Ylänne, Williams, & Wadleigh, 2009), newspapers (Martin, Williams, & O’Neill, 2009; Rozanova, Northcott, & McDaniel, 2006; Uotila, Lumme-Sandt, & Saarenheimo, 2010), animated films (Blakeborough, 2008; Robinson, Callister, Magoffin, & Moore, 2007), paintings (Wikström, 2000), song lyrics (Aday & Austin, 2000), and even birthday cards (Ellis & Morrison, 2005).

Previous analysis of the television portrayals of older people indicate that they are underrepresented in terms of the actual population. On television, older people and old age are mainly portrayed in a positive light; they are depicted as cognitively alert and in good physical health, and old age is presented as a phase of activity and productivity (Kessler, Rakozy, & Staudinger, 2004; Lien et al., 2009; Signorielli, 2004). However, older characters are often portrayed more akin to those in middle age, and the attributes of old age are mostly excluded (Kessler et al., 2004; Signorielli, 2004). For example, older people are often portrayed as still working (Kessler et al., 2004).

Advertisements, appearing in various media such as magazines and television, also tend to portray older people more positively than negatively (Ylänne et al., 2009; Zhang et al., 2006). The images of older people in photographs and advertisements are still rather permanent and standardized. Positive depictions include traits and characteristics like being happy, active, affluent, and influential. Negative depictions include traits and characteristics like physical frailty, vulnerability, and “otherness” (Whitfield, 2001).

Newspaper portrayals of older people have remained largely unexplored compared to the other media forms (Rozanova et al., 2006). However, newspaper portrayals of older people have been identified as more negative than in other media forms: loneliness is often presented as an inseparable part of old age (Uotila et al., 2010) and older people are presented as frail, infirm, and dependent (Fealy et al., 2012) as well as a burden and non-contributors to society (Martin et al., 2009). Thus, older people are portrayed as constituting a weak, passive “fourth age” (Rozanova, 2010; Uotila et al., 2010).

Cultural and societal factors influence the portrayals of older people and old age. As such, the portrayals of older people are different in Western and Eastern cultures (Lee, Kim, & Han, 2006; Raman, Harwood, Weis, Anderson, & Miller, 2008). Specifically in the USA, references to aging, older adulthood, and ill health seem to be slightly more common than in Asia, in part because advertisements for health products are also more common (Raman et al., 2008). In Eastern cultures, the overall salient societal respect for older people can be identified (Lee et al., 2006; Raman et al., 2008; Zhang et al., 2006). However, older people are still underrepresented in the media in terms of the actual population in different parts of the world (Kessler et al., 2010; Lee et al., 2006; Raman et al., 2008).

The portrayals of older people in a country’s media can also be influenced by its demographics and the specific details of its healthcare system. Finland is one of the first European nations to be facing the challenges of an aging population; by the year 2030, the proportion of the population aged 65 years and over is projected to be 25%, and for those aged 80 years and over, it will be 8% (Lanzieri, 2011). The healthcare system is tax-based, and the core values of equality, solidarity, and security are the basis of the Finnish universal social model, just as in the other Nordic countries. This philosophy is considered to go beyond the immediate needs of public-sector service provision (Björnsdóttir, Ceci, & Purkis, 2014; Greve, 2007; Nordic Council, 2012). Moreover, the provision of social and healthcare services is regulated and standardized: laws about care, for example, those concerning the status and rights of patients or the professional standards of personnel, apply to all public-sector services (Ministry of Social Affairs and Health, 2012). The Act on Supporting the Functional Capacity of the Older Population and on Social and Health Services for Older Persons (980/2012), several years in the making, came into effect in July 2013.

In summary, a society’s attitudes towards older people are illustrated in newspapers, but conversely, media portrayals have also been shown to affect people’s attitudes. The possible differences between the various portrayals that can be seen globally arise from the characteristics of the form of media and of the particular culture and system in each country. Thus, Finnish newspapers offer a new perspective for research on a global level as well as a national one. Because older people will be heavy users of health and social services in the future in Finland, it is important to study how they are viewed in society more generally, as this will ultimately influence the care that is provided to them, and thus their well-being.

## Aim

The aim of the study was to explore the portrayal of older people in Finnish society, as illustrated by newspapers. The research question was: how are older people perceived in society within the context of health? The context of health was selected because the ultimate goal is to enhance the well-being of older people by making current attitudes towards them visible.

## Method

This study applied ethnographic approach in textual reality in newspapers. For this purpose, culture is seen as a cognitive system where newspapers illustrate the ideational system of a society, such as the ideas, beliefs, and knowledge used when talking about older people and old age (Aamodt, 1982; Kim, 2010). Newspapers present these as “social facts” (Atkinson & Coffey, 2004), thus providing insight into the wider social world (Miller & Alvarado, 2005). Our analysis sought to theorize older people in this sociocultural context. Health was seen as the core of analysis from the perspective of nursing studies (Kim, 2010).

### Data collection

The data were collected from the three main, most popular Finnish daily newspapers: *Helsingin Sanomat*, *Aamulehti*, and *Turun Sanomat* (MediaAuditFinland, 2011, Table I); altogether, their readership covers 31% of the population over 15 years of age in Finland (Official Statistics of Finland, 2011). These newspapers are not politically committed, which is consistent with the majority of the newspapers in Finland. *Helsingin Sanomat* is a national newspaper and *Aamulehti* and *Turun Sanomat* are the leading papers in their respective markets in the main provincial towns (Jyrkiäinen, 2013). The data collection lasted 3 months (March to May 2012) and there were no major national debates at that time regarding older people and their health care.

**Table I:** Newspaper statistics and the number of selected articles.

			Newspapers (*n*)				Selected articles (*n*)				Types of the selected articles	
			
Newspaper	Audited circulation^a^	Readership^a^	March	April	May	In total	March	April	May	In total	Factual text (*n*)	Opinion text (*n*)
Helsingin Sanomat	365,994 (1st)	883,000 (1st)	31	28	30	89	9	13	11	33	12	21
Aamulehti	130,081 (2nd)	293,000 (5th)	31	28	31	90	8	8	13	29	18	11
Turun Sanomat	103,314 (3rd)	235,000 (6th)	31	28	30	89	11	13	15	39	19	20
In total	599,389	1,411,000	93	84	91	268	28	34	39	101	49	52

a MediaAuditFinland (2011); Readership: 2nd Ilta-Sanomat (tabloid), 3rd Iltalehti (tabloid), 4th Maaseudun Tulevaisuus (straight translation The Rural Future, covers news on agriculture and forestry as well as related businesses, and on rural enterprises and life in general in the countryside).

### Sample

All of the newspapers were published daily (*n*=268), except on certain holidays (Table I). The selection of newspaper articles for analysis was based on the following sampling strategy (Miller & Alvarado, 2005). One researcher (SK) read all of the newspapers and selected 133 articles for further examination based on the technical and content inclusion criteria (Table II). These initially selected articles were then read, following the selection criteria, by two researchers (SK, LS). Finally, 101 articles meeting the inclusion criteria were selected for analysis (Table I).

**Table II:** Technical and content inclusion and exclusion criteria for the article selection.

Inclusion criteria	Exclusion criteria
Technical criteria:	Technical criteria:
Daily newspaper	Supplement or specialized issue
Factual text (news, reports) on front page, domestic, foreign, financial, sports, culture or event section OR opinion text (leading article, column, pieces opposite the editorial page, comments, letters to the editor)	Photographs, pictures, advertisements, announcements, crime and birthday articles, cartoons
Content criteria: Article was selected if all the following criteria were realized:	Content criteria: Article was excluded if any of following criteria were realized:
Article considered older people over retirement age (>65 years) OR aging	Article considered age groups other than just people over retirement age (e.g., aging employees)
Article considered health (including sickness, ability to function, health promotion) in some way OR social and/or health services	Article considered issues other than health or social and/or health services
Article considered the above mentioned issue(s) in a broader and/or public context and justified it in some way	A single opinion about a certain situation or organization was expressed in the article

### Ethical considerations

In this study, a good scientific practice was followed (Finnish Advisory Board on Research Integrity, 2012). There was no need for ethical approval or permission to conduct the study because all the newspapers are publicly available. The newspapers in themselves were not analyzed as such and no comparisons between the newspapers were made. Direct quotations are here presented anonymously, even if names were included in the original articles. The original newspapers and selected articles are stored in an untouched form.

### Data analysis

**Figure 1:**
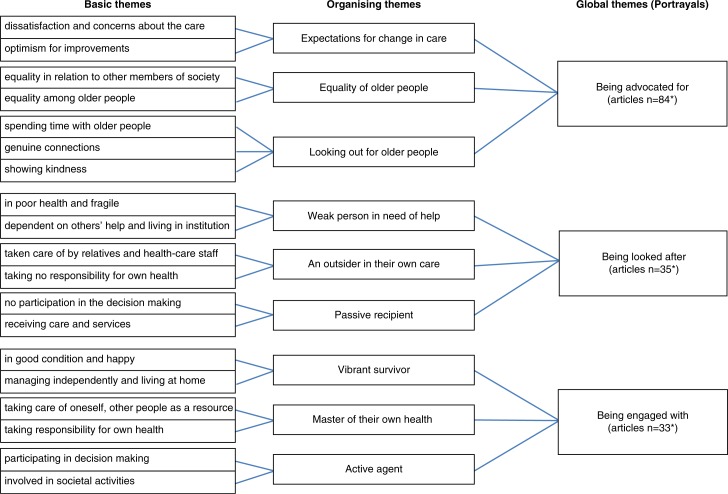
Portrayals about society’s attitudes towards older people. *It was possible for articles to be into multiple portrayals.

In the analysis, inductive (data-driven) thematic analysis (Braun & Clarke, 2006) was used as an appropriate technique for analyzing documents (Bowen, 2009). The first analytic phases were concerned with familiarizing ourselves with the data and coding. We conducted the coding incident by incident, by using the words from the articles to stay close to the data. The rest of the analytic phases covered generating and reviewing the themes which was an iterative process requiring movement back and forth between the coded data extracts and themes. Basic themes were first derived from the coded data extracts based on their similarities. Alike, basic themes were then classified as organizing themes and these organizing themes were brought together as global themes (Figure 1) (Attride-Stirling, 2001; Braun & Clarke, 2006; Hammersley & Atkinson, 2007). The basis of the conceptualization of the themes was the content of the articles. The analysis resulted in richer descriptions and more contentual variation for some themes. The findings are presented as global themes (portrayals).

### Rigor

The trustworthiness of the study was assured by using the criteria presented by Lincoln and Cuba (1985). One researcher (SK) concentrated on the data collection and the initial analyses, becoming familiar with the data at an in-depth level, and maintaining a sense of the broader picture. The research group met regularly to discuss the analysis and the next steps to be taken. The basic themes were generated individually by one researcher (SK). These were presented to the co-researchers and some parts of texts were processed together as an example to evaluate and ensure the consistency of the analysis. The whole research group was involved in generating, revising, and refining the organizing and global themes. For validation purposes, the preliminary findings were presented to a group of PhD candidates; no disagreement about the findings emerged. We present direct quotations below to enable readers to judge the procedure and findings.

Saturation was reached by including different types of text from various sections of the newspapers to obtain diverse data. Furthermore, articles were not excluded based on authors or interviewees, and thus the variety of the informants is broad; indeed, the fact that the number of representatives in certain informant groups is minimal can be seen as a snapshot of the reality at that time.

## Findings

### Description of the newspaper articles

Among articles selected for the study, 49 were factual texts and 52 opinions (Table I). For the factual texts, the interviewees were most often not older people themselves (Table III). However, older people and their relatives were asked about their personal opinions or experiences and they were often presented as examples of those affected by the matter being discussed. They were not asked to speak for all older people or give any rationales for their answers. Similarly, for the opinion pieces, older people and the relatives of older people rarely wrote for themselves (Table III).

**Table III:** Interviewees in and authors of the selected articles.

Interviewees/authors	Interviewees in the factual texts (*n*)	Authors of the opinion texts (*n*)
Older people	19	2
Relatives of older people	5	6
Social and healthcare professionals	22	8
Representatives of government and municipalities	41	3
Private persons (not identifiable otherwise)	0	14
Academics	9	2
Representatives of the third sector	7	3
Representatives of the care service companies	3	0
Editors and journalists	Not applicable	15
Representatives of the labor and employers’ organizations	3	3
In total	109	56

Three portrayals were identified as describing society’s attitudes towards older people: “being advocated for,” “being looked after,” and “being engaged with.” The first portrayal, “being advocated for” describes society’s attitudes towards older people through other people’s reactions without specifying what these older people are like. The second and the third portrayal, “being engaged,” with “being looked after,” concern society’s attitudes towards older people in terms of their personal characteristics, their residential environment, and their level of social participation as well as the issue of taking personal responsibility for health and the principal actors involved in their care.

### Being advocated for

The portrayal “being advocated for” consists of three organizing themes: the expectations for change in care, the equality of older people, and looking out for older people.

The first organizing theme, the expectations for change in care, was about dissatisfaction and concerns about the care and optimism for improvements. For the dissatisfaction and concerns about the care, the quality of care was experienced as unsatisfactory and there were doubts over whether the society is prepared for the necessary services for the increasing aging population. As one representative of the trade union for nurses stated: “Many surveys show that ordinary citizens do not want any savings in the care of the elderly. It is strange that these messages are not taken seriously” (HS b 28 March).

For optimism for improvements, the public attitudes were, however, more optimistic than pessimistic about the care of older people; indeed, many explicit, evidence-based actions had been taken to improve the care of older people. For example, the thoughts of one registered nurse were reported:

According to X there are clear indications that the elderly are eating better than before. She says that even though in the past the belief was that people just wither away and die this does not need to be the case. (TS 11 March)

The second organizing theme, the equality of older people, appeared in two ways: in relation to other members of society and among older people themselves. All older people were regarded as being entitled to fair treatment and specialized services within society, just like any other demographic (e.g., children, prisoners, or those with mental or physical disabilities). Furthermore, the downgrading of older people in relation to other members of society was unacceptable, as illustrated by the following comment: “After all, children in day care centers are not left indoors unattended with the assumption that their parents would come and take them outdoors during their lunch breaks” (HS 18 April).

The public attitude about older people was that it is important to secure equal access to an equal amount of services, providing the same quality at the same cost as other services across the country, to all older people. However, older people were also presented as a heterogeneous group with various needs, not only because of their chronological age, but also with differences in their health, ability to function, wealth, and sex. Consequently, there are sometimes conflicts when the needs of an individual cannot be taken into account because of the objective standards used in securing equal treatment for all older people. One close relative of an old person posed the following questions:

Near the place where I live there is a city-owned sheltered housing unit where many residents are fitter and younger than my mother and who have no problem moving about in groups both indoors and outdoors. Why have they secured a place in a sheltered housing unit? Is dementia always a requirement? (HS a 28 March)

On the other hand, sometimes a person’s individuality in “small,” ordinary, matters was taken into account, as the following comment from one music therapist highlights:

It is important to ask the patients what kind of music they would like to listen to or what songs they would like to sing …. According to X, it is of no use if the nurse liked Bach if the patient does not care about his music. (TS 11 April)

The third organizing theme, looking out for older people, was concerned with the need to spend time with older people, genuinely connecting, and showing kindness. All citizens were presented as having a part to play in the pursuit of a better old-age experience. Older people were not seen as being so different and each one of us should acknowledge them and help them on a daily basis. As a journalist and a relative of one older person pointed out:

The best way to help them (the veterans) is to do small things, such as chatting with them, taking them outdoors, and listening to what they have to say. (HS a 3 March)

The wanderings of my mother-in-law taught me that city dwellers do care. People who knew her took her by the hand and escorted her back home. People who did not know her wondered where an elderly lady wearing a bathrobe and a swimsuit was going. (HS 31 March)

### Being looked after

The portrayal “being looked after” consists of three organizing themes: a weak person in need of help, an outsider in their own care, and a passive recipient. In the first organizing theme, a weak person in need of help, the older person is characterized by a reduced ability to function described as poor physical condition and fragility manifested as a dwindling amount of strength and decreased movement. Furthermore, the older person can no longer manage on their own and is dependent on another’s help in many everyday situations. Thus, these older people commonly live in institutions. One official from the Regional State Administrative Agency stated: “People living in nursing homes are not bingo players but bedridden human beings requiring care on a 24-h basis” (AL 14 May).

In the second organizing theme, an outsider in their own care, close relatives and healthcare staff are the most active actors in the care of an older person; they are also the ones who take responsibility for the health of the older person because the older person has very limited self-care abilities. Close relatives participate in the care of an older family member differently. This can lead to conflicts between the relatives and healthcare staff: the healthcare staff expects close relatives to be committed to participating in the care of an older family member at home and in a care facility. One nurse from a sheltered home was reported as saying:

I work as a nurse in a sheltered home and I’m really amazed how little people contribute to making the everyday life of their elderly family members more pleasant …. Why can’t you take your close family members outdoors to give them some fresh air or take them to events or to a hairdresser? (HS 13 April)

Conversely, close relatives have been reported as feeling that they already have too great a responsibility for the care of their older family members. These relatives are also reported to feel that they are made to feel guilty for not participating enough in the care of their older family member or being too demanding with regard to their expectations about the type of care their family member should be receiving. The following comment was reported as a response to healthcare staff:

Close relatives have bad conscience and these matters keep us busy day and night. Surprisingly enough, we have time to do paperwork, monitor the well-being of our elderly family members, to take them to a dentist, and to remind staff of all kinds of matters. (HS 18 April)

In the third organizing theme, a passive recipient, participation in society’s activities for these older people is limited, and concentrated in just receiving social and healthcare services. Furthermore, they usually take only a limited, if any, part in the decision-making process concerning these services and even their own care. For example, one daughter wrote of her experiences concerning her mother:

The aim is to get the elderly to 24-hour sheltered housing as quickly as possible …. As the elderly gradually become less alert and are less and less able to take care of themselves, the bureaucratic complexities of different types of care are highlighted …. The elderly were like pawns: evening wash at 5 pm, followed by evening meal after which they went to their own rooms to stare the empty walls around them. (HS b 22 April)

### Being engaged with

The portrayal “being engaged with” consists of three organizing themes: vibrant survivor, master of their own health, and active agent. In the first organizing theme, vibrant survivor, the older person is characterized by an ability to function, based on a generally good physical condition, and being in a happy state of mind. Furthermore, such a person is independent and needs or wants very little help for handling their everyday activities. Thus, these older people live in their own house or in another place that they consider to be their home, like sheltered housing.

In the second organizing theme, master of their own health, older people are often active actors in their care and use other people merely as a resource to keep in shape if they require support. They take responsibility for maintaining their own health by participating in different activities offered for these purposes. An older person taking part in an exercise group told about her life: “Here I am, lifting weights. The exercise makes my arms stronger so that I’m able to cook my own food. X says that she does all her household chores herself” (TS a 20 April).

In the third organizing theme, active agent, these older people participate in society’s activities. At the least, these older people have opportunities to make decisions in matters that concern them. Participation can also occur outside personal settings and manifest, for instance, in senior club activities or municipal planning. The following example comes from the municipal level: “Elderly people living in the area can today suggest their own ideas about the future services for the area …. The event is intended for the elderly, their close relatives, and people working with the elderly” (AL 29 March).

## Discussion

This study explored how newspapers in Finland portray older people in society. The portrayals of older people in the newspapers in this study contrast somewhat with the findings in previous studies, which reported mostly negative portrayals (Martin et al., 2009; Rozanova, 2010; Uotila et al., 2010). Based on the portrayal “being advocated for,” all older people despite what they are like and their care were considered important in society. This portrayal fits with the idea of the Nordic model, where everyone, including vulnerable groups, are seen as equally valuable members of society and entitled to equal access to social and healthcare services (Nordic Council, 2012). On the other hand, this could be an indication of one form of ageism, as it fails to acknowledge the diversity of older people in society, and treats them as a homogenous group (Phelan, 2008). Furthermore, despite the generally positive attitudes towards older people, the somewhat negative presentation of the public perception of older people who are “being looked after” is a significant finding as it could be indicative of unequal and thus negative attitudes towards this particular group (Rozanova, 2010).

As in the study by Rozanova (2010), we found different portrayals of older people in terms of their health or the idea of successful aging. In contrast to her findings, however, we did not find frailer older people being blamed for their condition, but their poor health was nevertheless highlighted, which could be indicative of ageist attitudes (Phelan, 2008). Our findings about older people who are “being engaged with” resonate with the ones concerning older home-dwelling people who find doing things important and an investment in maintaining their own health and well-being (Söderhamn, Dale, & Söderhamn, 2013). When aging successfully means staying engaged in activities (Rozanova, 2010), it is important to explore whether older people who participate in activities experience the expectations to do so, and the pressures to keep in good health. We did not find suggestions that older people are depicted as a burden on society despite the economically challenging times and the aging population requiring services (Martin et al., 2009).

It is noteworthy that the ability to function was the one determining factor we found for the different portrayals of older people, and not chronological age. In this study, a newspaper article was included in the analysis if it included people aged 65 or over. The age of 65 is the most common official age of pension entitlement in the countries of the Organisation for Economic Cooperation and Development (OECD, 2011a), and thus is commonly used as the definition of the beginning of old age. However, there is no general agreement on the chronological age at which a person becomes old. As our findings also indicate, other socially constructed meanings are in fact more significant than chronological age, such as the roles assigned to older people.

The division of different portrayals of older people based on their residential environment may also be a reflection of the care arrangements in Finland, where institutionalized care is more common than it is in other countries, for example, Japan and Norway. This may be because, in Finland, a larger number of people have more severe conditions or live in remote areas where home-based care options may be limited (OECD, 2011b). Therefore, a residential facility is more commonly used to assume and define the condition and capability of the older person. Similar connotations concerning the “right” place of residence have previously been identified in Iceland, where there is a discussion on whether the applied measures for determining the place of residence also take into account the individual needs of older persons (Björnsdóttir et al., 2014).

This division between different portrayals of older people also reflects the perceptions of family involvement in the care of older people. Previously, a cross-country study concerning eight European countries differing in demographic composition and terms of long-term care revealed that there were obvious differences between countries for informal caregiving and supportive actions (Hallberg et al., 2013). The findings of our study are in agreement with those from previous Nordic studies showing that the society was predominantly seen as the main source of care, but that close relatives were still seen as an important source of assistance (Björnsdóttir et al., 2014; Haavio-Mannila et al., 2009; Haberkern & Szydlik, 2010). Quite the contrary, in southern and some central European countries, filial obligations are accompanied by a rudimentary care infrastructure with a relatively high prevalence of intergenerational care being the care arrangement preferred by the majority (Haberkern & Szydlik, 2010).

The different portrayals of older people in terms of their level of societal participation were not in line with the idea behind the Nordic model, where the society is expected to create opportunities for all people to have an active social life and to take part in the decision-making process (Nordic Council, 2012). Indeed, no suggestions were presented for how older people who are “being looked after” participate in society. On the other hand, active participation is not a universal aspiration. Older people are content without taking an active part in the community, even if explicit opportunities present themselves. Exercising the right “not to take part” is suggested to be the strongest determinant of nonparticipation, rather than specific constraints (Curry & Fisher, 2013). However, it is important that society and healthcare professionals respond to the realization that even the most vulnerable older people can participate in society if they choose to.

All three portrayals illustrate different kinds of care practices towards older people. The portrayal of “being engaged with” describes autonomous older people who are using healthcare professionals merely as a resource for maintaining their health. Despite the positive premise of the portrayal of “being advocated for,” it is possible that older people are seen as incapable of taking care of, and speaking for, themselves. This is supported by the fact that older people rarely appeared as informants in the articles. This is in line with the findings from other studies demonstrating that older people were underrepresented in the media, respective of their actual population (e.g., Kessler et al., 2010). Resultantly, the voices of older people themselves remain marginal, and possibly unheard. Similarly, when considering how “being looked after” is portrayed, close relatives and healthcare staff were the principal actors in the care of older people. In both of these portrayals, there are features of paternalism, commonly regarded as ageism in professional practice (Phelan, 2008). However, paternalism can often be difficult to distinguish from advocacy (Zomorodi & Foley, 2009). To overcome the possibility of paternalistic care practices, ensuring that there are ways for all older people to be heard, is one step towards older people becoming a more inherent part of the decision-making processes concerning them.

### Methodological considerations

In this study, we focused on health, and social and healthcare services. Our methodology was designed to avoid a biased perspective as the data were collected from multiple newspapers. Furthermore, the data is unique, because it was originally created without any influence by the researchers and thus can be regarded as independent in that sense (Bowen, 2009).

Newspapers as data, present, in their own right, a valuable documentary version of real life (Atkinson & Coffey, 2004). An analysis of newspaper portrayals constitutes one way of obtaining insights into wider society and its practices that are otherwise difficult to acquire (Miller & Alvarado, 2005). However, our findings cannot be used in isolation as evidence about the general public’s attitudes towards older people; rather, they support other inquiries that provide real-life illustrations of the attitudes of people in a certain period of time. Thus, data collection over a longer time period or repeated data collection could have been beneficial in terms of supporting the findings, and the requirement that ethnographic methodology should provide a lengthy exposure to the field being studied (Atkinson & Coffey, 2004).

The findings of this study are only applicable to the Finnish, and possibly with caution to the Nordic, context at the studied time. However, the findings resonate for many Western countries and thus these findings may also provide a useful contrast for other researchers from other countries. We achieved confirmability by writing both scientific and reflective notes during the research process (Lincoln & Cuba, 1985).

## Conclusions

This study identified three portrayals of older people from newspaper articles, which can be understood as illustrations of the attitudes towards older people in the Finnish society at that time. In relation to the cultural context, our findings are in accordance with the attributes of Nordic culture and society, but they also share similarities with some results from previous international studies that consider the media portrayals of older people.

Overall, all older people and their care were regarded as important in this society, suggesting that the societal attitudes towards older people are generally positive. However, there were also indications of negative attitudes based on the suggestions of paternalistic attitudes towards older people. Moreover, although older people, and their care, are regarded as important in society, different groups of older people are viewed differently within society, which may possibly lead to inequality.

While noting that healthcare professionals’ attitudes are not independent from or outside of general societal attitudes, healthcare professionals need to understand the influence of media portrayals on their own attitudes towards older people and, moreover, their ways of providing care (Phelan, 2011). In graduate and advanced education, healthcare professionals should be supported in detecting the influence of these media portrayals on their own attitudes and actions. Working with the media should also be a concern of healthcare professionals. They can use the recommendations formulated for journalists (Dahmen & Cozma, 2009) when contributing to public discussions to avoid accidentally reinforcing less favorable attitudes towards older people and aging.
